# From theory to 'measurement' in complex interventions: Methodological lessons from the development of an e-health normalisation instrument

**DOI:** 10.1186/1471-2288-12-69

**Published:** 2012-05-17

**Authors:** Tracy L Finch, Frances S Mair, Catherine O’Donnell, Elizabeth Murray, Carl R May

**Affiliations:** 1 Institute of Health and Society, Newcastle University, Baddiley-Clark Building, Richardson Road, Newcastle-upon-Tyne, NE2 4AX, England; 2 Institute of Health and WellBeing, University of Glasgow, 1 Horselethill Road, Glasgow, G12 9LX, Scotland; 3 Research Department of Primary Care and Population Health, University College London, Upper Floor 3, Royal Free Hospital, Rowland Hill Street, London, NW3 2PF, England; 4 Faculty of Health Sciences, Building 67, University of Southampton, Highfield, Southampton, SO17 1BJ, England

## Abstract

**Background:**

Although empirical and theoretical understanding of processes of implementation in health care is advancing, translation of theory into structured measures that capture the complex interplay between interventions, individuals and context remain limited. This paper aimed to (1) describe the process and outcome of a project to develop a theory-based instrument for measuring implementation processes relating to e-health interventions; and (2) identify key issues and methodological challenges for advancing work in this field.

**Methods:**

A 30-item instrument (Technology Adoption Readiness Scale (TARS)) for measuring normalisation processes in the context of e-health service interventions was developed on the basis on Normalization Process Theory (NPT). NPT focuses on how new practices become routinely embedded within social contexts. The instrument was pre-tested in two health care settings in which e-health (electronic facilitation of healthcare decision-making and practice) was used by health care professionals.

**Results:**

The developed instrument was pre-tested in two professional samples (N = 46; N = 231). Ratings of items representing normalisation ‘processes’ were significantly related to staff members’ perceptions of whether or not e-health had become ‘routine’. Key methodological challenges are discussed in relation to: translating multi-component theoretical constructs into simple questions; developing and choosing appropriate outcome measures; conducting multiple-stakeholder assessments; instrument and question framing; and more general issues for instrument development in practice contexts.

**Conclusions:**

To develop theory-derived measures of implementation process for progressing research in this field, four key recommendations are made relating to (1) greater attention to underlying theoretical assumptions and extent of translation work required; (2) the need for appropriate but flexible approaches to outcomes measurement; (3) representation of multiple perspectives and collaborative nature of work; and (4) emphasis on generic measurement approaches that can be flexibly tailored to particular contexts of study.

## Background

Advancements in new technologies of health and medical care – and in their social organisation - promise to benefit the health and well-being of patients and society. However, getting new technologies into practice beyond the context of research projects that demonstrate the (clinical) efficacy or effectiveness of new practices and procedures remains a problem. Researchers are now investing much effort in understanding and resolving issues of ‘implementation’ in relation to health care interventions and practices, and this is reflected in a fast growing field of ‘implementation science’. Understanding the science behind implementation processes has also become an important concern for healthcare policy and practice. Following Linton [1]:

‘Implementation involves all activities that occur between making an adoption commitment and the time that an innovation either becomes part of the organizational routine, ceases to be new, or is abandoned (…) [and the] behavior of organizational members over time evolves from avoidance or non-use, through unenthusiastic or compliant use, to skilled or consistent use. (p 65)’

There is a vast literature on implementation in service organisations [2], however efforts at implementing new technologies and practices remain problematic. The gap between research evidence and practice remains wide [3], and concerns about the large numbers of ‘pilot’ studies of new interventions that never lead to sustainable services are repeatedly expressed [4]. This is particularly the case for ‘e-health’ technologies - defined as practicing and delivering health care using information and communication technology [5] - despite significant promises for improving health care quality and efficiency [6].

In attempting to address such problems of implementation, the application of theory to designing health care interventions [7], planning and evaluating them [8][9,10], and developing effective strategies for their implementation [11] offers much potential.

However, obstacles to the use of theory for such purposes are numerous, and include the identification of relevant and useful theoretical perspectives from the huge body of literature on implementation that spans diverse academic disciplines (for example, psychology, sociology, business, healthcare management). Such theoretical diversity includes approaches that emphasise attitudes and behaviours [8,12,13]; diffusion and adoption of innovations through social networks [14]; and Science and Technology Studies (STS) approaches [15,16] that emphasise technology design and its relations with human actors. Reviews such as those of Greenhalgh and colleagues [2] (of literature relevant to the diffusion of innovations in service organisations) and Grol and colleagues [8] (of theories useful for planning and studying initiatives for improving patient care) begin to address this difficulty by mapping the terrain of implementation theories that may be useful for guiding both intervention development and approaches to implementation, and summarising their key processes and emphases.

Advances in theory-based intervention development and implementation have been made particularly with regard to changing healthcare professionals’ behaviour and practice to facilitate the uptake of evidence-based-practice strategies [7,17]. Drawing on psychological theories of behaviour, Michie and colleagues [17] explicitly set out to develop theory-based explanations of factors that affect professional practice in a format that would be accessible to non-academic users, and associated work has included guidance for designing questionnaires based on the Theory of Planned Behavior [18]. Models focused on psychological theory however, tend to over-emphasise the personal agency of individuals and underplay the importance of context. For example, implementation failures are often attributed to slow behaviour change by professionals, when there are likely to be other good and predictable socio-organisational reasons for such failure [19]. Nonetheless, such approaches show promise in facilitating the uptake of new interventions and/or ways of working, particularly where the roles and actions of individuals in making an implementation ‘effective’ are an appropriate focus for implementation efforts.

We would argue, however, that in practice, many interventions being implemented in healthcare settings are subject to more complex influences than those known to directly affect the behaviour of individuals. New practices get taken up and become ‘workable’ due to a complex interplay between features of the intervention/practice itself, the actions of individuals involved in the process, and aspects of the physical and social environment in which implementation activities are undertaken. Normalization Process Theory (NPT) [20,21] approaches the problem of implementation with a view to understanding such dynamics. It emphasises the processes by which new technologies and practices become *normalised,* focusing on the work that this requires of people working both individually and collaboratively. What really matters here is the extent to which new technologies and practices can – and do – become *embedded* in both the contexts in which they are to be used, and in the everyday practices of the individuals whose work is affected by these innovations. NPT is concerned with the generative processes [22] that underpin three core problems: *implementation* (bringing a practice or practices into action); *embedding*, (when a practice or practices may be routinely incorporated into the everyday work of individuals and groups); and *integration* (when a practice or practices are reproduced and sustained in the social matrices of an organization or institution). In NPT it is postulated that practices become routinely embedded in social contexts as the result of people working, individually and collectively, to enact them, and that the production and reproduction of a practice requires continuous investment by individuals to carry action forward in time and space. There are four sets of processes that characterise different kinds of ‘normalisation work’, and which require particular kinds of investments from individuals and organisations [20,21]:

Coherence: the process of sense-making and understanding that individuals and organisations have to go through in order to promote or inhibit the routine embedding of a practice to its users. These processes are energized by investments of meaning made by participants.

Cognitive participation: the process that individuals and organisations have to go through in order to enrol individuals to engage with the new practice. These processes are energized by investments of commitment made by participants.

Collective action: the work that individuals and organisations have to do to enact the new practice. These processes are energized by investments of effort made by participants.

Reflexive monitoring: the informal and formal appraisal of a new practice once it is in use, in order to assess its advantages and disadvantages and which develops users’ comprehension of the effects of a practice. These processes are energized by investments in appraisal made by participants.

A considerable body of research now supports NPT as an adequate and useful theory for explaining processes of the normalization of practices associated with complex interventions. This evidence spans diverse settings in which new technologies and practices have been the focus of its application, such as telecare [23], e-health [24,25], clinical decision support systems [26], teledermatology [27], infertility management [28], maternity services [10]and the management and treatment of depression [29,30].

The development of structured tools for assessing implementation processes, which take account of this complex interplay between interventions, individual actions, and context, would represent an advance in applying theory to understand and address implementation problems in practice. Existing assessment tools that focus on organisational factors relevant to ‘readiness’ for interventions in healthcare [31-33] do not adequately reflect the complexity of normalisation processes as characterised by the NPT – for example, the dynamic and iterative relationships between the types of work involved in making sense of a new practice, enacting it (collectively) and appraising its outcome and value. They are therefore limited in the extent to which they offer practical ways of *facilitating* implementation processes in ways that lead to the embedding of new practices within contexts of use.

A further challenge for the development of theory-based measures that capture the complexity of implementation activities concerns the various ways in which *outcomes* of such activities may be defined. In contrast to psychological theories of implementation behaviour, which focus on explaining and/or quantifying individuals’ uptake of a new practice, NPT focuses on more subtle – and gradual – processes, such as ‘embedding’, ‘integrating’ and ‘normalisation’. NPT does not offer a ‘definition’ of the term ‘normalisation’, for it can be appropriately used to refer to a process or a ‘state’, depending on the context and the frame of reference – that is, for the most part ‘normalisation’ is considered to be an ongoing cycle of activity aimed at making a new practice ‘fit in’ with the work of individuals and their context of practice, but when a practice ceases to be ‘new’ or no longer requires additional effort, it may be framed as having become ‘normalised’. Further work needs to be done to develop ways of defining and measuring *outcomes* of efforts to implement new practices, that reflect the complexity and context-dependent nature of what it means to have ‘successfully’ or ‘effectively’ implemented a new practice.

Thus the development of structured assessment tools for understanding the complex processes involved in integrating complex interventions, including e-health [34], into practice remains a priority. Recently, theory-based tools for assisting implementers in planning and ‘thinking through’ particular interventions with reference to the social and organisational contexts in which they are to be implemented have been offered [35,36]. Although promising however, such tools do not provide *measurements* to be used during implementations to assess progress towards successful implementation (however defined by stakeholders). Such tools would offer the potential to identify (and quantify) problems with an implementation during the process, but so far work in this area remains limited.

The objective of this study then was to advance work on translating theory into structured assessment instruments for research and practical purposes in these contexts, by drawing on the findings of a study [24] that undertook the development and preliminary testing of a Technology Adoption Readiness Scale (TARS) for measuring normalisation processes in the context of e-health service interventions. This paper therefore aims to (1) describe the process and outcome of a project to develop a theory-based instrument for measuring processes involved in the implementation of e-health interventions; and (2) identify key issues and methodological challenges for further advancing work in this field. First however, a fuller explanation of the theoretical development of NPT is required.

### Normalization process theory: Theoretical development

NPT was initially developed as an applied theoretical model to assist clinicians and researchers to understand and evaluate the factors that inhibit and promote the routine incorporation of complex healthcare interventions in practice. Since then, it has been developed as a middle-range theory of socio-technical change [20], which characterizes the mechanisms involved in the embedding of practices within their immediate and broader social contexts.

The development of NPT [37] focused on addressing two key criteria for theory to be ‘useful’: that it must be both adequately described and fit for purpose. Thus, the theory has been developed to offer transparent and transferable explanations for the phenomena of interest (processes of embedding new practice and ways of working) revealed by empirical investigation [38,39]. In doing so, we have followed sociological approaches to theory building [22,40,41] to undertake four kinds of conceptual work required to make a theory ‘fit for purpose’: describing, explaining, making knowledge claims, and investigating observed phenomena (see Table 1: Requirements of Theory).

**Table 1:** **Requirements of a Theory (from May et al. 2007**[42])

	
** *1. Accurate description* **	A theory must provide a taxonomy or set of definitions that enable the identification, differentiation, and codification of the qualities and properties of cases and classes of phenomena.
** *2. Systematic explanation* **	A theory must provide an explanation of the form and significance of the causal and relational mechanisms at work in cases or classes of the phenomena defined by the theory, and should propose their relation to other phenomena.
** *3. Knowledge claims* **	A theory must lead to knowledge claims. These may take the form of abstract explanations, analytic propositions, or experimental hypotheses. They may also map relations with other phenomena that are believed to possess similar qualities and properties.
** *4. Investigation* **	A theory must be testable. Such tests may be abstract (i.e. formal logical representations, simulations, or thought experiments); or concrete (empirical investigations).

Considerable work has been undertaken to critique NPT in terms of its potential for describing key processes that underpin the success or otherwise of implementation, and to ensure that NPT’s core constructs can be operationalised in a stable and consistent way by multiple user constituencies, including testing out NPT in qualitative studies of a variety of practices and in a diverse range of contexts [10,23-30]. Recent work has also extended the practical utility of NPT for a wide range of academic and non-academic users. An online ‘users’ manual’ for NPT ( http://www.normalizationprocess.org), that provides descriptions, guidance on use of the theory, and applied examples, along with work to frame NPT as a tool for *designing, developing and implementing* complex interventions [9] and make NPT accessible to diverse user groups who are interested in understanding and solving practical problems of implementation.

The development of good practice for designing and administering structured instruments to *assess the processes of normalization* described and explicated in the formal specification of the theory is the next step for further extending the utility of NPT. In terms of enhancing the NPT’s ‘fitness for purpose’, this is important for facilitating *investigation* as a key component of theory (Table 1). The development of NPT derived ‘assessment’ measures would represent a step beyond current work undertaken with NPT to operationalise it as a tool for planning interventions [9,35], towards exploring investigative questions about the theory’s scope for use in *predicting* – or more appropriately providing assessment of ‘*potential for achieving’*[21]– the normalization of complex interventions in practice.

### Development of technology adoption readiness scale (TARS)

An instrument development study was undertaken as part of a larger study that used a multi-method approach to understanding barriers to the uptake and integration of e-health into healthcare professionals’ practice [43]. The TARS study aimed to develop a structured instrument to measure *processes* of normalisation in relation to the routine use of a specific e-health system. As NPT is the basis for the instrument, these normalisation processes are seen to reflect staff perceptions of factors related to the collaborative work required for the normalisation of particular e-Health systems *in a given context.* The primary purpose of this instrument then was to enable users to quantify a range of processes proposed by the NPT to contribute to the successful normalisation of a new intervention – in this case, e-health. As such, the instrument could be used both by practitioners charged with implementing an e-health intervention (and thus used in a ‘diagnostic’ capacity for identifying and resolving problems early on in an implementation), and by research teams or practitioners undertaking service evaluations (thus as an evaluative tool). Although the ultimate aim of a programme of work we are undertaking on measure development based on NPT is to develop ‘predictive’ tools based on the theory, development of an instrument for this purpose was beyond the scope of this study.

This project was undertaken in two stages, each of which is described here in turn. The first stage was the development of the instrument and the second stage was a preliminary test of the utility of the instrument in two different NHS settings in which staff were using particular e-health systems. The focus of this project was on development rather than the empirical determination of psychometric properties, thus the final discussion in this paper will focus primarily on the processes and experiences of translating empirically derived theoretical constructs into structured tools and the implications of this for undertaking applied assessments in health care settings.

### Phase 1: Item development and conceptual validation

In this phase, we aimed to draw on the NPT to develop a comprehensive set of general items –TARS items - reflecting factors affecting the routine use of e-health ready for application in specific settings.

## Methods (phase 1)

The first step was understanding the key ‘assumptions’ of NPT and identifying implications and challenges for developing measures based on the theory. Table 2 outlines the key considerations regarding this process, which will be returned to in the discussion. Rather than prescribing specific methodological processes, this preliminary analysis served as a general frame of reference to guide the development of TARS.

**Table 2:** Key challenges for developing NPT based measures

**NPT: Key assumptions**	**Implications and challenges**
Individuals’ own perceptions of a new practice are important and worth of assessment	General psychological principles of measurement are relevant and useful
Assessment of individuals’ perceptions of *the work involved in a new practice *, rather than their own intentions or actions is required	Direct implications for how questions are framed Outcomes for measurement are likely to be more complex than those based on individual behaviour Outcomes of interest will be specific to the kind of work required and the particular context in which it is conducted
Understanding work as ‘collaborative’ requires assessment of all groups of individuals who are affected by a new practice	Sampling and recruitment of appropriate professional groups is key Requires in-depth understanding of the different roles of constituent groups and their working contexts Likelihood of requiring different versions of an instrument for constituent groups
As a theory of socio-technical change, change over time is a key focus of NPT	Direct implications for how questions are framed, and raises possible alternatives for approaches to assessing impact or making comparison between competing practices Timing of assessments is a key consideration

### Item generation

The TARS items were developed using three sources of knowledge about factors that affect the use of e-health: *theoretical* knowledge as represented by the NPT; *empirical* knowledge, in the form of findings of a meta-review of e-health being conducted as a related project [24]; and *expert* knowledge obtained using an expert survey (described below).

At the time the study commenced, we were working with the Normalisation Process Model (NPM) [44], therefore the bulk of the questions developed for inclusion stemmed from the NPT’s ‘Collective Action’ construct (see below for brief descriptions, and elsewhere [20,42] for accounts of the theory development process). In NPT, the key constructs of NPM remain of central importance but as processes underlying a more general construct of Collective Action that relates firmly to the ‘enactment’ stage of an intervention.

Contextual Integration (CI): the degree to which the proposed e-health system fits (or integrates) with the overall goals and structure of the organisation (context), as well as the capacity of the organisation to undertake the implementation.

Relational integration (RI): the way in which different professional groups relate to each other, and how well the proposed e-health initiative fits (or integrates) with existing relationships, as well as the degree to which it promotes trust, accountability and responsibility in inter-group relationships.

Interactional workability (IW): the degree to which the e-health system enables (or impedes) the work of interactions between health professionals and patients – e.g. a consultation.

Skill set workability (SSW): the degree to which the e-health initiative fits with existing working practices, skill sets, and perceived job role.

Item construction began by translating the theoretical constructs into plain language statements, each of which having a single and comprehensible meaning. For example, the construct of ‘*contextual integration’* included the statement that a factor affecting the normalisation of a new technology is ‘…… the extent to which organizational effort is allocated to an e-Health system in proportion to the work that the system is intended to do.’ Such statements were simplified, for example, to ‘sufficient organisational effort has gone into supporting the system’ and ‘the rewards of using the system outweigh the effort’. This process resulted in 23 items for rating which, after critical peer review, were increased to a final set of 27 rating items to be included in the expert survey.

### Expert survey

An online survey of experts was conducted to (a) test the face validity of items intended for inclusion in the final item set and (b) collect data about the perceived relative importance of individual items. The 27-item set was pilot-tested as a live link by members of the project advisory group (n = 5), resulting in minor refinements (shown in Table 3). In the survey, participants were asked to rate the *importance* of each item to the routine use of e-Health, using a scale in which 0 = not at all important; 1 = some importance; 2 = moderate importance; 3 = very important; 4 = extremely important; with the option of choosing 'don't know'.

**Table 3:** Descriptive analysis of results of Expert survey

		**Considerations**	**Decision**	**Final Item**
Q1	Allocation of financial resources to the system	Ranked in top half of table. Correlates with q.2 (0.527) and q. 18 (0.531)	retain	Allocation of financial resources to the system
Q2	Allocation of organizational effort to the system	Third highest mean rating score. Correlates with q.1 (0.527).	retain	Allocation of organizational effort to the system
Q3	Impact of the system on existing ways of working	Ranked no 1 in importance. No r’s > 0.5.	retain	Impact of the system on existing ways of working
Q4	Balance of effort against rewards of using the system	Ranked 5^th^. Doesn’t correlate well with any other item	retain	Balance of effort against rewards of using the system
Q5	Impact of the system on individual’s perceptions of autonomy in their work	Mid-table in importance ratings. Correlates with q.9 (r 0.573).	retain	Impact of the system on individual’s perceptions of autonomy in their work
Q6	level of co-operation required from others *within *the organisation, in using the system	Ranked 8^th^. Correlates with q.7 (0.560).	Combine 6 and 7	Level of co-operation required by others in using the system
Q7	level of co-operation required from others *outside *the organisation, in using the system	Correlates with q.6 (0.560), but most correlations near zero. (ranked 5^th^ from bottom)		
Q8	Additional workload created by the system	Ranked 4^th^ in importance. No r’s above 0.5, but approaching that on q. 26 and 27.	retain	Additional workload created by the system
Q9	Impact of the system on allocation of work between individuals	Correlates with q.5 (r 0.573).	retain	Impact of the system on allocation of work between individuals
Q10	Compatibility of the system with existing skills	Ranked mid-table. Correlates with q.11 (0.519)	retain	Compatibility of the system with existing skills
Q11	Obtainability of new skills required to use the system	Ranked 11^th^. Correlates with q.10 (0.519). Several significant (but low) correlations with other items.	retain	Obtainability of new skills required to use the system
Q12	Impact of the system on individuals’ perceptions of personal liability	Ranked 3^rd^ from bottom. Correlates with q. 17 (r .564) & 18 (r .569). Correlations < but approaching 0.5 for q. 13 & 14.	exclude	
Q13	Individuals’ own confidence in the safety of using the system	Ranked mid-table. High r (0.725) with q. 14. Correlates with q. 18 (0.565). Approaches 0.5 with q.12.	Combine 13 and 14	Individuals’ own confidence in the safety of using the system
Q14	Individuals’ confidence in the safety of *others’ *use of the system	Ranked least important. High r (0.725) with q. 13, and correlates with q.18 (0.531). Approaches 0.5 with q.12.		
Q15	Individuals’ perceptions of the efficiency of using the system	Ranked mid-table. No correlations > 0.5.	Retain	Individuals’ perceptions of the efficiency of using the system
Q16	Impact of the system on the distribution of ** *responsibilities * ** between individuals	Ranked in bottom half. No correlations > 0.5.	Retain	Impact of the system on the distribution of responsibilities between individuals
Q17	Impact of the system on individuals’ beliefs about their accountability for their work	Ranked near bottom. High r with Q.18 (0.806). Correlates with q. 12 (r .564)	retain	Impact of the system on individuals’ beliefs about their accountability for their work
Q18	Impact of the system on individuals’ beliefs about ** *others’ expectations * ** of their accountability for their work	Ranked second bottom. High r with Q.17 (0.806). Correlates with q. 12 (r .569), 13 (0.565) and q.14 (0.531).	Exclude question	
Q19	Availability of ** *technical expertise * ** in using the system	Ranked in top half. Correlates with q.21 (0.557) & 25 (0.581).	retain	Availability of technical expertise in using the system
Q20	Availability of an ** *evidence base * ** about the clinical effectiveness of the system	Ranked in bottom half. High r with Q.21 (0.721). Also r 0.619 with Q.24.	Combine 20 and 21	Availability of evidence about the clinical effectiveness of the system
Q21	Availability of ** *users’ * ** knowledge of the clinical effectiveness of the system	Ranked in bottom half. High r with Q.20 (0.721). Correlates with q.19 (0.557), q. 24 (0.517) & q.25 (0.514).		
Q22	How flexibly the system can be used for conducting work	Ranked in top half. Correlates with q.23 (0.533).	retain	How flexibly the system can be used for conducting work
Q23	Perceived impact of the system on ** *ways of working with * ** patients	Ranked 6^th^ in importance. Correlates with Q.22 (0.533). & q.25 (0.586).	Retain	Perceived impact of the system on ways of working with patients
Q24	Perceived impact of the system on ** *outcomes * ** for patients	Ranked mid-table. Correlates with Q.20 (0.619) & q.21 (0.517).	retain	Perceived impact of the system on outcomes for patients
Q25	Perceived impact of the system on ** *communication * ** with patients	Ranked mid-table. Correlates with q.19 (0.581), q. 21 (0.514) & q.23 (0.586).	Exclude (covered in q 23)	
Q26	Perceived impact of the system on the ** *amount of time * ** spent with patients	Ranked in top half. Approaches 0.50 with q.8. & q.25.	retain	Perceived impact of the system on the amount of time spent with patients
Q27	Ease of using the system	Ranked second highest in importance. Doesn’t correlate > .05 with any item.	retain	Ease of using the system

The sample was defined as authors of published reviews of e-health, drawing on papers included in the scoping review, and supplemented with additional searching of relevant fields (eg. telecare, telemedicine) to develop a sufficient sampling frame. A database of 308 potential respondents with (unverified) email addresses was produced. Authors were invited via email to take part in the survey, and were sent personalised links for response tracking. Non-responders were sent up to two reminders, approximately 10 days apart.

## Results (phase 1)

A total of 63 participants completed the expert survey out of 252 invitations (24% response) that were presumed to be received (subtracting invitations returned as ‘undeliverable’). Sample characteristics are presented in Table 4. Details of ratings for the item set are reported elsewhere [24] (and available as Additional File 1), but in general, items were highly endorsed by the survey participants as important factors affecting the routinisation of e-health systems.

**Table 4:** Sample characteristics of expert survey participants

**Location of Residence**	**%**		
USA	37		
UK	27		
Canada	13		
Europe (excluding Scandinavia)	10		
Australia/New Zealand	8		
Scandinavia	6		
**Research background**			
Medical	32		
Social science	24		
Informatics	21		
Nursing	11		
Economics	2		
Health Services Research	5		
Non-specific	6		
**Sex**			
Male	59		
Female	41		
**E-health domain**	**Mostly (%)**	**Partly (%)**	**Not at all (%)**
Management Systems	29	46	25
Communication Systems	44	32	24
Computerised decision support systems	14	38	48
Web based Information Resources	22	29	49

Preliminary descriptive analysis was undertaken to make decisions about excluding or combining existing items, analysing each item in terms of (i) the mean rating of importance for that item, and (ii) any correlations between the item and other items in the set (correlations of r > 0.5). The results of this decision analysis are presented in Table 3. Items that were highly correlated with other items were either discarded or re-written into a single item, particularly where importance ratings were relatively low. This process reduced the 27 items to 21.

Participants in the Expert survey were invited to suggest (using free-text) any factors they felt to be particularly important and which they believed had not been covered in the item set. Analysis of these free-text comments made by (n = 31) survey participants resulted in the eventual inclusion of five new items about contextual integration issues (Q.5-9 in Table 5). Peer review (amongst the project team) resulted in further revisions, notably the addition of three items to reflect the NPT’s constructs of coherence, cognitive participation, resulting in a final set of 30 generic TARS items ready for adapting for use in specific contexts.

**Table 5:** Final set of TARS items

**Q.**	**NPT**	**Final Tars Items**
1.	CA-CI	The ehealth system is adequately resourced financially
2.	CA-CI	Sufficient organizational effort has gone into supporting the ehealth system
3.	CA-CI	The ehealth system is a different way of working
4.	CA-CI	The rewards of using the ehealth system outweighs the effort
5.	CA-CI	Government policy initiatives are supportive of this ehealth system
6.	CA-CI	This ehealth system is technically and organisationally compatible with other systems and agencies that we are required to work with
7.	CA-CI	This ehealth system fits in with the priorities and challenges of our organisation
8.	CA-CI	This organisation has a culture that is supportive of change
9.	CA-CI	There is a culture in this organisation of involving staff in planning and development
10.	CA-SSW	Using the ehealth system makes me feel autonomous in my work
11.	CA-SSW	Using the ehealth system requires co-operation with other staff
12.	CA-SSW	The workload involved in using the ehealth system is manageable
13.	CA-SSW	In using the ehealth system, the allocation of work between individuals is appropriate
14.	CA-SSW	The skills I have are appropriate for using the ehealth system
15.	CA-SSW	The skills needed to use the ehealth system are easily learned
16.	CA-RI	I have confidence that using the ehealth system does not put patients at risk
17.	CA-RI	Using the ehealth system is an efficient use of time
18.	CA-RI	In using the ehealth system, responsibilities are divided between individuals appropriately
19.	CA-RI	In using the ehealth system, I understand my accountability for my work
20.	CA-RI	In using the ehealth system, I understand my liability for my practice
21.	CA-RI	Technical back-up in using the ehealth system is available if I need it
22.	CA-RI	I believe there is good evidence about the clinical effectiveness of using the ehealth system
23.	CA-IW	There is some flexibility in how the ehealth system can be used
24.	CA-IW	Using the ehealth system leads to positive outcomes for patients
25.	CA-IW	Using the ehealth system involves the right amount of time spent with patients (on the telephone)
26.	CA-IW	In using the ehealth system, the quality of professional and patient interaction is good
27.	CA-IW	The ehealth system is easy to use
28.	Coherence	The staff who work here have a shared understanding of what the system is for and how it is to be used
29.	Cognitive Participation	The staff here are committed to making the system work
30.	Reflexive Monitoring	There are ongoing mechanisms for monitoring and appraising how this ehealth system is used

### Phase 2: Testing TARS items in specific health contexts

#### Methods (phase 2)

This phase tested the utility of TARS for assessing normalisation processes in relation to specific e-health systems, using convenience samples in two NHS contexts. These sites were chosen because (i) specific e-Health systems were in use by health professionals, and (ii) the two sites reflected different levels of ‘normalisation’ of e-health. At Site 1, use of the e-health system (community nurses using Personal Digital Assistant technology) was relatively new, and provided an opportunity to use the TARS items in a context where e-health was still in the experimental stages for some users. At Site 2, the entire organisation is based on e-health systems – so staff could be expected to have greater experience of e-Health and over a longer time.

The factor statements developed in Phase 1 were translated into directional statements and given a 7 point response scale eliciting level of agreement in relation to the e-health technology being assessed in that context. The scale of responses was anchored at either end with ‘strongly disagree’ and ‘strongly agree’ with non-labelled interim points. Explanatory text and demographic questions varied slightly between sites. Following the set of TARS rating items, two additional questions were included to assess: (i) participants’ perceptions about whether the system was not at all, partly, or completely in routine use; and (ii) their perceptions about the likelihood of it becoming routine (on a 5 point scale: definitely not; probably not; possibly; probably will; definitely will). Although the complexity of developing outcome ‘measures’ to represent the concept of normalisation has already been noted (and was not the focus of this study), these questions were included to represent perceptions of the current state of normalisation of the e-health technology, for the purpose of exploring the utility of the TARS items that were developed to represent processes contributing to normalisation.

In both sites, the survey was conducted electronically using a commercial provider ( http://www.surveymonkey.com). Site contacts facilitated participant recruitment and management of response rates via reminders. At both sites, two reminders were issued following the original invitation (at intervals of 10 – 14 days), which increased response rates. The research team did not have direct access to staff details and email addresses (as our ethical approval for the project did not extend to accessing staff personal details).

Data were analysed descriptively, using frequency tables to visually assess the distributions for ratings elicited using the scales. As responses on individual items were in many cases skewed and non-normally distributed, non-parametric cross-tab analysis with Pearson’s Chi Square statistic was used to explore differences in perceptions relating to TARS items according to perceived level of routinisation of the e-health system. For these analyses, new categorical variables were created by combining rating points. For Site 1, responses to the TARS items were dichotomised into groups indicating non-agreement (responding 0 strongly disagree −3 neutral midpoint) and those responding with various levels of agreement (rating 4–6). At Site 2 (with a larger sample size and different spread of responses), TARS item responses were trichotomised as follows: Disagreement (0–2); neutral or some agreement (3 or 4); and moderate to strong agreement (5 or 6).

## Results (phase 2)

At Site 1, 46/243 participants completed the survey (19% response rate). At Site 2, 231/1351 (17% response rate) completed the survey sufficiently for inclusion in the analysis.^a^It should be noted that response rates are approximate and conservative, as calculation is based on total number of staff emailed an invitation to participate. These rates do not reflect adjustment for reasons for non-participation such as absence from work, or failed delivery of emails, as such information was not available to the researchers. It should be noted that response rates are approximate and conservative, as calculation is based on total number of staff emailed an invitation to participate. These rates do not reflect adjustment for reasons for non-participation such as absence from work, or failed delivery of emails, as such information was not available to the researchers. Sample characteristics for both sites are presented in Table 6, and Table 7 presents frequencies for the combined categorical variables, to indicate item responses. Tables 8 and 9 present the significant results for the Chi Square analyses for each site respectively, and ‘n’ denotes sample sizes for the different cells within each analysis (which differ from frequencies presented in Table 7 because ‘don’t know’ responses were excluded from these analyses on a per item basis). These analyses indicated that, for a number of items, stronger positive endorsement was indicated by participants who perceived e-health to be routine, thus supporting the NPT. For Site 1, significant differences between groups perceiving e-health as ‘partly routine’ compared with ‘completely routine’ were evident for 12 out of the 30 items. For the these items, the pattern of relationship is such that those who perceived the e-health system to be completely a routine part of their work were more likely to agree than not agree with the statements about the system, or to show a higher proportion within the group responding with agreement (ie overall, they indicated more positive responses). Here, the strongest significant differences occurred on two of the Contextual Integration items ‘this organization has a culture that is supportive of change’ and ‘this e-Health system fits in with the priorities and challenges of our organization’, along with the Coherence item ‘the staff who work here have a shared understanding of what the system is for and how it is to be used’.

**Table 6:** Sample characteristics for Phase 2 participants (Site 1 and Site 2)

	**Site 1% (n)**		**Site 2% (n)**
**Age groups:**		**Age groups:**	
<25	0 (0)	<25	9 (20)
25-34	4 (2)	25-34	20 (47)
35-44	24 (11)	35-44	32 (73)
45-54	59 (27)	45-54	33 (75)
55+	13 (6)	55+	7 (15)
**Sex**		**Sex**	
Male	0 (0)	Male	14 (32)
Female	100(46)	Female	86 (199)
**Working role:**		**Working role:**	
Community Enrolled Nurse	0 (0)	Call handlers	47 (109)
Community Staff Nurse	28 (13)	Nurse advisors	24 (56)
District Nursing Sister/Charge Nurse	61 (28)	Team leaders	9 (21)
Practice Development Nurse	9 (4)	Health information advisors	3 (7)
Senior Nurse	2 (1)	Other	16 (38)
**Time working in role:**		**Time working in role:**	
<2 years	7 (3)	< 1 year	15 (36)
2 to < 5 years	22 (10)	1 year to 23 months	10 (23)
5 to <10 years	28 (13)	2 years to 47 months	20 (45)
10 years plus	30 (14)	4 years to 71 months	16 (36)
Did not specify	13 (6)	6 years +	16 (37)
		Did not specify	23 (54)
**Time using e-Health system**		(Time using not assessed for Site 2)	
no months of use	9 (4)		
some but <3 mths	20 (9)		
4 or 5 mths	9 (4)		
6 mths but <12	20 (9)		
1 yr but <2 yrs	22 (10)		
2 years +	22 (10)		
**Perceived routinisation of e-Health**		**Perceived routinisation of e-Health**	
Not at all	0 (0)	Not at all	1 (2)
Partly	68 (30)	Partly	17 (35)
Completely	32 (14)	Completely	83 (174)

**Table 7:** TARS items: Frequencies for combined categorical variables

		**Site 1**				**Site 2**				
**Item**		**Disagree/neutral (0–3)**	**Agree (4–6)**	**DK**	**N**	**Disagree** **(0–2)**	**Neutral/Some agree (3–4)**	**Stronger Agreement (5–6)**	**DK**	**N**
1	The ehealth system is adequately resourced financially	21	12	13	46	16	60	44	111	231
2	Sufficient organizational effort has gone into supporting the ehealth system	21	21	4	46	25	91	66	49	231
3	The ehealth system is a different way of working	16	22	8	46	10	59	105	56	230
4	The rewards of using the ehealth system outweighs the effort	22	15	8	45	14	95	72	44	225
5	Government policy initiatives are supportive of this ehealth system	13	23	8	44	12	67	44	106	229
6	This ehealth system is technically and organisationally compatible with other systems and agencies that we are required to work with	36	3	6	45	46	86	44	53	229
7	This ehealth system fits in with the priorities and challenges of our organisation	25	16	3	44	18	112	77	21	228
8	This organisation has a culture that is supportive of change	20	23	0	43	25	106	81	10	222
9	There is a culture in this organisation of involving staff in planning and development	28	16	0	44	75	89	46	19	229
10	Using the ehealth system makes me feel autonomous in my work	31	9	2	42	43	92	66	23	224
11	Using the ehealth system requires co-operation with other staff	17	27	2	46	29	113	74	14	230
12	The workload involved in using the ehealth system is manageable	25	18	3	46	20	109	86	15	230
13	In using the ehealth system, the allocation of work between individuals is appropriate	19	16	9	44	26	96	66	42	230
14	The skills I have are appropriate for using the ehealth system	13	31	1	45	7	73	139	11	230
15	The skills needed to use the ehealth system are easily learned	16	27	2	45	24	112	85	9	230
16	I have confidence that using the ehealth system does not put patients at risk	13	28	3	44	22	92	99	15	228
17	Using the ehealth system is an efficient use of time	7	36	1	43	23	91	104	13	231
18	In using the ehealth system, responsibilities are divided between individuals appropriately	7	36	6	43	26	100	66	35	227
19	In using the ehealth system, I understand my accountability for my work	14	29	2	45	7	59	148	8	222
20	In using the ehealth system, I understand my liability for my practice	21	20	1	44	9	61	134	19	223
21	Technical back-up in using the ehealth system is available if I need it	14	24	2	45	46	95	62	26	229
22	I believe there is good evidence about the clinical effectiveness of using the ehealth system	10	19	0	43	26	129	74		229
23	There is some flexibility in how the ehealth system can be used	18	19	9	45	57	98	48	28	231
24	Using the ehealth system leads to positive outcomes for patients	21	20	5	45	17	119	72	22	230
25	Using the ehealth system involves the right amount of time spent with patients (on the telephone)	27	16	12	43	43	104	54	28	229
26	In using the ehealth system, the quality of professional and patient interaction is good	18	18	14	44	21	121	69	19	230
27	The ehealth system is easy to use	26	16	2	43	22	112	85	8	227
28	The staff who work here have a shared understanding of what the system is for and how it is to be used	25	15	3	44	22	107	86	11	226
29	The staff here are committed to making the system work	21	9	5	43	15	101	93	15	224
30	There are ongoing mechanisms for monitoring and appraising how this ehealth system is used	21	10	16	45	7	89	83	50	229

**Table 8:** Site 1 Chi Square analysis of agreement with TARS items by perception of level of routinisation

		**Partly routinen (non-agree, agree)**	**Completely routinen (non-agree, agree)**	**χ**
4	The rewards of using the e-Health system outweighs the effort	23 *(17, 6)*	14 *(5, 9)*	5.268*
5	Government policy initiatives are supportive of this e-Health system	26 *(13, 13*)	11 *(0, 11)*	8.479**
7	This e-Health system fits in with the priorities and challenges of our organization	27 *(21, 6)*	14 *(3, 11)*	12.061***
8	This organization has a culture that is supportive of change	30 *(18, 12)*	14 *(1, 13)*	10.870***
16	I have confidence that using the e-Health system does not put patients at risk	28 *(12, 16)*	14 *(1, 13)*	5.570*
17	Using the e-Health system is an efficient use of time	29 *(20, 9)*	14 *(5, 9)*	4.289*
19	In using the e-Health system, I understand my accountability for my work	29 *(7, 22)*	14 *(0, 14)*	4.036*
21	Technical back-up in using the e-Health system is available if I need it	28 *(11, 17)*	14 *(1, 13)*	4.725*
27	The e-Health system is easy to use	28 *(17, 11)*	14 *(3, 11)*	5.775*
28	The staff who work here have a shared understanding of what the system is for and how it is to be used	27 *(18, 9)*	14 *(2, 12)*	10.124***
29	The staff here are committed to making the system work	25 *(12, 13)*	14 *(2, 12)*	4.433*
30	There are ongoing mechanisms for monitoring and appraising how this e-Health system is used	15 *(8, 7)*	14 *(2, 12)*	4.887*

**Table 9:** Site 2 Chi Square analysis of agreement with TARS items by perception of level of routinisation

**Item:**	**Disagree** **N (NP, C)**	**Neutral or some agreement** **N (NP, C)**	**Moderate or strong agreement** **N (NP, C)**	**χ**
Sufficient organizational effort has gone into supporting the e-Health system	23 (30, 11)	83 (52, 50)	59 (17, 39)	7.757*
The e-Health system is a different way of working compared with other parts of the NHS	9 (0, 7)	51 (63, 28)	98 (37, 66)	9.818**
This organization has a culture that is supportive of change	24 (9, 13)	98 (72, 47)	70 (19, 40)	6.868*
The skills I have are appropriate for using the e-Health system	7 (10, 2)	69 (55, 31)	123 (35, 67)	12.714**
In using the e-Health system, I understand my accountability for my work	6 (8, 2)	53 (50, 24)	135 (42, 74)	10.918**
In using the e-Health system, I understand my liability for my practice	9 (20, 3)	54 (36, 28)	124 (44, 70)	16.503***
I believe there is good evidence about the clinical effectiveness of using the e-Health system	25 (24, 9)	119 (54, 58)	65 (22, 33)	7.109*
The staff who work here have a shared understanding of what the system is for and how it is to be used	19 (22, 8)	98 (52, 50)	78 (26, 42)	6.576*
There are ongoing mechanisms for monitoring and appraising how this e-Health system is used	6 (5, 4)	79 (74, 45)	78 (21, 51)	6.196*

* denotes significance level of p < 0.05; **p < .01; ***p < .001

At Site 2, nine TARS items indicated significant differences in responses between participants perceiving different levels of routinisation (Table 9). These results suggested that compared with those who feel that e-health has already become ‘completely routine’, those for whom it hasn’t become routine were less likely to agree that sufficient organisational effort has gone into supporting the system; and less likely to show strong agreement (rather than being neutral or some agreement) that: e-health is a different way of working; that the organisational culture is supportive of change; that they understand their own accountability and liability; and that there are ongoing mechanisms for monitoring and appraising how e-health is used. The group for whom e-health was not yet a completely routine part of their practice were also more likely to disagree that there is good evidence of clinical effectiveness of the e-health system, and that there is a shared understanding of what the system is for and how it is to be used. Here, the strongest differences between groups were evident on items relating to liability, accountability and appropriateness of skills.

Together, the results from both sites suggest that the ratings made on the instrument items are related to participants’ perceptions of how routinely the e-health systems are being used in their practice contexts.

## Discussion

This paper has set out to (1) describe the process and outcome of a project to develop a theory-based instrument for measuring processes involved in the implementation of e-health interventions based on Normalization Process Theory; and (2) identify key issues and methodological challenges for further advancing work in this field.

The practical output of this study was the development of the TARS instrument, which was intended to enable researchers and practitioners to quantify a range of processes proposed by the NPT to contribute to the successful normalisation of e-health, either as a ‘diagnostic’ tool or for evaluation purposes. Developing TARS required considerable ‘translation work’, both in terms of the methodological implications of the theory’s underlying theoretical assumptions (Table 2), and from theoretical constructs into specific questions. To develop a set of assessment items with good face validity, multiple sources of information were collected and utilised including theoretical specifications of NPT (and its underlying empirical basis), the perspectives of academic experts in e-health implementation, and primary qualitative data concerning professionals’ views of implementation and integration of e-health in the NHS [24]. Whilst the expert survey (Phase 1) endorsed the proposed items as reflecting important factors affecting the potential for e-health to become a routine part of working practices (and suggested further items about contextual integration), health professionals themselves indicated greater emphasis on practice-based issues concerning benefits, particularly to patients, and workload management. Representation of different kinds of ‘expertise’ thus ensures that research instruments being developed for use in practice contexts are ‘fit for purpose’. In this study, we were focused primarily on health professionals using e-health in their day to day work, but even within this focus there were important differences between the roles and experiences of staff in relation to the e-health systems we studied, that affected their capacity to answer all of the questions. Although the questions included in the instrument were developed drawing on multiple sources of stakeholder input in general, this finding does raise concerns about the level of face validity achieved for the specific groups within our samples. We suggest that in using an instrument such as TARS, continued work on ensuring face validity of questions at the level of the participants within the local setting of use is required. We must also acknowledge that the results presented in this study are limited by focusing primarily on nurses as a professional group. In other studies, for example, it will also be important to consider assessments from the perspectives of a more diverse range of medical and healthcare professionals, managers and/or implementers [25], or indeed patients [45] . This study thus highlights the collaborative nature of health care work, and the importance of ensuring that multiple-stakeholders’ perspectives [46] are incorporated into the development of tools to assess implementation processes in these contexts.

As one of the first studies to use the NPT in quantitative research, this study aimed to progress work on NPT towards statistical investigation of relationships between implementation processes and outcomes in terms of ‘normalisation’. Although only tests of associations (rather than causality) between normalisation processes and outcomes were possible in this study, ratings of normalisation processes differed between groups holding different perceptions of whether or not the e-Health systems in the respective study sites had become part of routine practice. The two study sites themselves differed – both in terms of the technology being implemented (mobile electronic devices to facilitate community nursing versus computerised decision support services) and the level of progress towards the technology being considered ‘normal’, so differences between them in terms of which items related to perceptions of normalisation would be expected. Although preliminary, however, these finding lend support to assessing the potential predictive value of the TARS in prospective, longitudinal studies. Furthering work on the predictive utility of TARS – and NPT more generally – will however require flexible approaches to identifying and specifying ‘outcome’ measures. The process undertaken in this study demonstrates that ‘normalisation’ is highly context-dependent, relating to the practice itself, the environment in which it is operating, and the different groups of individuals that relate to it. As such, NPT does not provide any particular definition of ‘normalisation’ for the purpose of measurement as an outcome variable for quantitative studies, and designers of studies based on NPT to assess outcomes will need to develop study-specific measures based on what outcomes are relevant, and which are likely to be multiple and include both subjective (self-report) and objective (eg. usage data) measures. For example, just some normalisation ‘outcomes’ that might be considered are: level of use; increasing use over time; amount of shift from one practice to another; disappearance of a previous practice; reported acceptability of a practice; or measures of quality of work stemming from use of the practice. The development of approaches to measuring such outcomes will require not only developing and testing quantitative measures, but also further qualitative investigation about how people make judgements about whether or not a new practice can be considered ‘normalised’, and how that may or may not have happened.

This project aimed to develop a simple structured research instrument that could be used in other contexts. However, the process of considering the many possible ways to frame questions about processes involving change, demonstrated that use of tools such as TARS in other research context will require highly flexible and adaptive approaches to ensure that questions are framed appropriately to reflect the stage of implementation/use of the new technology or practice being studied. Here, we chose to frame questions as likert type statements about the e-health technology of interest and elicit respondents’ agreement with those statements, but in other situations it might be preferable to frame questions in a multitude of ways, such as: eliciting expectations of a technology planned but not yet used, inviting direct comparisons between key aspects of one type of technology/practice against another (eg. ‘X is a better way of working than Y’), or even assessing the perceived impact of the technology/practice over time (eg. ‘The impact of X on [practice] has been....). Although not intended at the outset of this study, the set of TARS items framed as ‘factors’ in the format in which they were presented for eliciting ratings of perceived relative importance (ie without reference to any direction of effect, as presented in Table 3) could be used for the development of research instruments that include questions framed according the specific objective of the study. This consideration may prove challenging for further validation of the TARS items as ‘an instrument’, but also offers a range of opportunities for practical use of the tool in assessing staff perceptions of issues that this study has shown to be important for the normalisation of e-health.

In relation to NPT, the study described in this paper has also contributed to theory development. It has successfully achieved the development of a set of quantitative questions that can be used to assess staff perceptions of processes relevant to the normalisation of e-health with reference to underlying aspects of the constructs within the Collective Action component of NPT, along with single items for assessing perceptions relating to the NPT constructs of coherence, cognitive participation, and reflexive monitoring. This development process, which included gathering and incorporating views from diverse sets of academic and professional stakeholders, challenged our thinking about the constructs, and the multiple interpretations that could be made about their meaning. In part, the processes described here contributed directly to the expansion of the theory from the NPM to the NPT as currently presented (see elsewhere [20,42] for detailed description). This process has continued beyond this study [36], and is likely to continue as the theory is used, tested and challenged for a variety of purposes.

Despite such limitations, the study offers preliminary support for the conceptual distinctions between and within the constructs of the NPT (particularly with respect to the Collective Action construct), and for the potential predictive potential of items in the instrument with respect to normalisation outcomes (as demonstrated by associations between NPT processes as represented by the TARS items and perceived normalisation of e-health in the contexts of study). Although the TARS instrument does not represent balanced coverage of NPT in its entirety, the key underlying assumptions of the theory as a whole – such as the focus on the collaborative nature of work required of a practice-based intervention – remain constant across the developmental shift from NPM to NPT and thus the methodological challenges and issues described in this paper are of enduring relevance. In relation to the TARS study, the emphasis on the ‘collective action’ component for framing data collection was appropriate, as we were undertaking assessments focused on the ‘enactment’ stage of e-health implementations. However, to further develop the TARS instrument – and to develop measures of NPT that more comprehensively cover the wider frame of implementation activity that spans stages of conceptualising (coherence), engagement of individuals (cognitive participation), and reflection/evaluation (reflexive monitoring) – more longitudinal research will be needed.

This study was focused primarily on instrument development rather than formal validation, however key limitations are worth noting. Despite considerable effort by the research team to maximise response rates, achieved rates were lower than expected. It is difficult to consider the implications of the response rates achieved, as the rates themselves are a ‘worst estimate’, as true response rates (ie in terms of percentages participating out of those who received and read the invitation) could not be calculated due to limited access to information. Reliance on key contacts at survey sites (who were helpful but already working under pressure) also limited the timing and frequency of reminders that could be achieved, and thus the need for greater researcher control over access to research participants must be emphasised. Also, selection of sites for data collection in this study was necessarily pragmatic, and access was negotiated well in advance of the instrument being developed and ready for data collection (as is often the case with applied research). Given that the study sites already had at least some level of adoption of e-Health technology, the scope for prospectively assessing the predictive value of the instrument items in terms of normalisation outcomes was not possible in this study, but should be the objective of further studies where assessment of perceptions can be undertaken prior to, during and after the implementation of a new practice-based initiative. In relation to health technology in general, the challenges of assessing new technologies in practice contexts are recognised [47] but worth emphasising here.

### Implications

In highlighting valuable lessons for theory-based instrument development, the study advances knowledge within the field of implementation science. The processes involved in implementing complex interventions are exactly that – complex. NPT has been built from over a decade of observation and analysis of the complex interplay of the structural, organisational, social, and individual factors that affect the ways in which new practices become (or do not become) embedded in routine practices and the contexts in which they are enacted. Such theoretical complexity presents challenges for the development and validation of ‘simple’ measures that can be used generically across contexts that differ qualitatively in ways that reflect the reality of health care service settings. However, the research described in this paper supports the observation of others [7,8] that this is a challenge that must be embraced as a means of facilitating the effectiveness and uptake of health care interventions in practice.

The findings of this study suggest four key recommendations for developing and assessing theory-derived measures of implementation processes useful for assessing complex healthcare interventions in practice. Firstly, careful consideration must be given to the underlying assumptions of the chosen theory itself, and to the considerable translation and validation work likely to be required (drawing on multiple sources of evidence) for the identification of key concepts and their appropriate expression as simple questionnaire-style items. Secondly, identification – or rather development- of appropriate measures of implementation (or normalisation) ‘outcomes’ is key to the practical utility of theory-derived measures for such assessments, but this is highly context-dependent and thus requires tailored development within specific study (or practice) contexts, for example through conducting preparatory (and qualitative) assessment of what it would mean for a particular intervention to be considered ‘normalised’ within that context. Thirdly, a comprehensive understanding of implementation and normalisation processes in any given context requires adequate multiple perspective assessments that are sensitive to the varied contributions of different professional (or other) groups working individually and collaboratively, and which reflect good understanding of the roles of such individuals and the contexts in which they conduct their work. Finally, we suggest that in undertaking theory-based assessments of this kind, it must be recognised from the outset that approaches to measurement must themselves be ‘fit for purpose’ and as such are unlikely to be achieved entirely by standardised measures developed for use across diverse settings. Thus, consideration should be given to the development of research instruments that come with guidance on how they can be applied flexibly according the objectives of the research study and specific contexts of use [18].

## Conclusion

Understanding the processes by which new technologies and practices can become normalised in health care settings – so that we can improve approaches to implementation - remains an important challenge for academics, policy makers, health care managers and practitioners. This study extended work on Normalization process Theory (NPT) towards tests of predictive utility of the theory by developing an instrument to assess normalisation potential in relation to e-health. We suggest that pursuit of the development of generic tools and measures for these purposes – such as the TARS instrument described here - is a useful starting point. However, the practical utility of theory-derived research instruments for measuring implementation and normalisation processes can only be fully realised through research and development activity that is focused on providing guidance for the operationalisation and adaptation of such measures for use in the contextually diverse environments in which health care work is conducted. We suggest that this study represents the beginning of a very complex journey.

## Endnote

^a^It should be noted that response rates are approximate and conservative, as calculation is based on total number of staff emailed an invitation to participate. These rates do not reflect adjustment for reasons for non-participation such as absence from work, or failed delivery of emails, as such information was not available to the researchers.

## Competing interests

The authors declare that they have no competing interests

## Authors' contributions

TLF conducted data collection and statistical analysis, and drafted the manuscript. TLF, CRM & FSM conceived of the study, TLF coordinated the study, and all other authors participated in its design and helped to draft the manuscript. All authors read and approved the final manuscript.

## Pre-publication history

The pre-publication history for this paper can be accessed here:

http://www.biomedcentral.com/1471-2288/12/69/prepub

## Supplementary Material

Additional file 1 **Table S1.**Means, Standard Deviations and Frequencies of importance ratings (Expert survey) Click here for file
