# Supplementary material for: From theory to 'measurement' in complex interventions: Methodological lessons from the development of an e-health normalisation instrument
Source: BMC Med Res Methodol. 2012 May 17;12:69. doi: 10.1186/1471-2288-12-69 (PMC3473304; doi:10.1186/1471-2288-12-69)
Supplement: Additional file 1 — Table S1.Means, Standard Deviations and Frequencies of importance ratings (Expert survey) [file 1471-2288-12-69-S1.docx]

**Supplementary Table: Means, Standard Deviations and Frequencies of importance ratings (Expert survey)**

|  |  |  |  |  | **Frequencies (% are approx double)** | | | | | | |
| --- | --- | --- | --- | --- | --- | --- | --- | --- | --- | --- | --- |
|  |  | **N*** | **Means** | **Sds** | **1** | **2** | **3** | **4** | **5** | **8 DK** | **Total ans** |
| 3 | Impact of the system on existing ways of working | 49 | 4.57 | .677 |  |  | 5 | 11 | 33 | 1 | 50 |
| 9 | An organizational culture of involving staff in planning and development | 47 | 4.57 | .677 |  |  | 2 | 14 | 31 | 2 | 49 |
| 15 | Compatibility of the system with existing skills | 46 | 4.57 | .677 |  | 1 | 11 | 13 | 21 | 3 | 49 |
| 21 | Impact of the system on individuals' perceptions of liability | 45 | 4.57 | .677 | 1 | 3 | 4 | 14 | 23 | 3 | 48 |
| 27 | Perceived impact of the system on the quality of the interaction between professionals and patients | 45 | 4.57 | .677 |  |  | 6 | 15 | 24 | 3 | 48 |
| 6 | The integration of the e-Health system with other systems and agencies that it must relate to for the conduct of work | 47 | 4.53 | .620 |  |  | 3 | 16 | 28 | 4 | 51 |
| 12 | Level of co-operation required by others in using the system | 47 | 4.53 | .620 |  | 1 | 5 | 15 | 26 | 3 | 50 |
| 18 | Individuals' perceptions of the efficiency of using the system | 48 | 4.53 | .620 |  | 1 | 5 | 19 | 23 | 2 | 50 |
| 24 | How flexibly the system can be used | 46 | 4.53 | .620 |  |  | 3 | 13 | 30 | 2 | 48 |
| 30 | The commitment of individuals to making the system work | 46 | 4.53 | .620 |  |  | 5 | 17 | 24 | 2 | 48 |
| 2 | Allocation of organizational effort to the system | 48 | 4.50 | .684 |  |  | 5 | 14 | 29 | 3 | 51 |
| 8 | An organizational culture that is supportive of change | 48 | 4.50 | .684 |  |  | 3 | 12 | 33 | 2 | 50 |
| 14 | Impact of the system on allocation of work between individuals | 44 | 4.50 | .684 | 1 | 2 | 9 | 14 | 18 | 6 | 50 |
| 20 | Impact of the system on individuals' beliefs about their accountability for their work | 47 | 4.50 | .684 |  | 2 | 5 | 16 | 24 | 2 | 49 |
| 26 | Perceived impact of the system on the amount of time spent with patients | 43 | 4.50 | .684 |  | 1 | 5 | 16 | 21 | 5 | 48 |
| 32 | The balance between the needs of individual users, other users of the system, and the organisation itself | 47 | 4.50 | .684 |  |  | 9 | 14 | 24 | 1 | 48 |
| 4 | Balance of effort against rewards of using the system | 49 | 4.37 | .755 |  |  | 8 | 15 | 26 | 2 | 51 |
| 10 | The level of autonomy that the organization has in terms of commissioning services | 45 | 4.37 | .755 |  |  | 8 | 19 | 18 | 5 | 50 |
| 16 | Learnabiliity of new skills required to use the system | 48 | 4.37 | .755 |  | 1 | 4 | 17 | 26 | 2 | 50 |
| 22 | Availability of technical support in using the system | 47 | 4.37 | .755 |  |  | 3 | 11 | 33 | 1 | 48 |
| 28 | Ease of using the system | 47 | 4.37 | .755 | 1 |  | 1 | 14 | 31 | 1 | 48 |
| 5 | The supportiveness or otherwise of the broader context in relation to e-Health | 39 | 4.18 | .885 |  | 1 | 9 | 11 | 18 | 11 | 50 |
| 11 | Impact of the system on individual's perceptions of autonomy in their work | 44 | 4.18 | .885 |  | 1 | 7 | 15 | 21 | 6 | 50 |
| 17 | Individual's own confidence that using the system does not put patients at risk | 47 | 4.18 | .885 |  | 1 | 3 | 7 | 36 | 2 | 49 |
| 23 | Availability of evidence about the clinical effectiveness of the system | 47 | 4.18 | .885 | 1 |  | 8 | 10 | 28 | 1 | 48 |
| 29 | The existence of a shared understanding of what the system is for and how it is to be used | 45 | 4.18 | .885 |  |  | 7 | 18 | 20 | 2 | 47 |
| 1 | Allocation of financial resources to the system | 48 | 4.10 | .951 | 2 |  | 7 | 21 | 18 | 3 | 51 |
| 7 | How well the e-Health system fits in with priorities and challenges of the organization | 48 | 4.10 | .951 |  | 1 | 5 | 15 | 27 | 3 | 51 |
| 13 | Additional workload created by the system | 48 | 4.10 | .951 | 2 | 2 | 10 | 10 | 24 | 2 | 50 |
| 19 | Impact of the system on the distribution of responsibilities between individuals | 45 | 4.10 | .951 |  | 2 | 15 | 16 | 12 | 5 | 50 |
| 25 | Perceived impact of the system on outcomes for patients | 45 | 4.10 | .951 |  | 1 | 3 | 15 | 26 | 2 | 47 |
| 31 | The existence of ongoing mechanisms for monitoring and appraising how the system is used | 45 | 4.10 | .951 |  | 1 | 5 | 15 | 24 | 3 | 48 |
